# Risk factors associated with invasive fungal disease post-liver transplantation

**DOI:** 10.1016/j.bjid.2026.105886

**Published:** 2026-07-03

**Authors:** Larissa Nunes de Almeida Gouveia, Maristela Pinheiro Freire, Bruno Graciano Ponce Romeo, Julia Rezende, Carolina Devite Bittante, Luis Fernando Aranha Camargo, Wellington Andraus, Luiz Augusto Carneiro D’Albuquerque, Edson Abdala, Alice Tung Wan Song

**Affiliations:** a Hospital das Clínicas da Faculdade de Medicina da Universidade de São Paulo (FMUSP), São Paulo, SP, Brazil; b Hospital Israelita Albert Einstein, São Paulo, SP, Brazil

**Keywords:** Liver transplantation, Invasive fungal disease, Risk factors, Mycoses, Survival

## Abstract

**Background:**

Invasive Fungal Infections (IFI) are significant causes of morbidity and mortality after liver transplantation. Infections occurring within the first 90-days post-transplant are considered early, with *Candida* species, *Aspergillus*, and *Cryptococcus* being the primary agents of early IFI. Various factors have been identified in studies of risk factors for IFI after liver transplantation, with differing results between studies and transplant centers. The main objective of this study was to identify risk factors associated with invasive fungal disease and mortality within the first 100-days post-liver transplantation in two Brazilian centers.

**Methods:**

A retrospective multicenter case-control study was conducted in two Brazilian liver transplant centers.

**Results:**

66 cases of IFI and 198 controls were analysed. Intrabdominal site infection was the most prevalent, in 34 cases (46%) and non-albicans *Candida* species were the main etiologies, and the most used prophylactic antifungal was fluconazole. In multivariate analysis, antifungal prophylaxis (OR = 0.397, 95% CI 0.167‒0.941) and deceased donor (OR = 0.312, 95% CI 0.109‒0.893) were protective factors, while renal replacement therapy (OR = 4.104, 95% CI 1.744‒9.965) and reoperation (OR = 5.368, 95% CI 2.739‒10.521) were independent risk factors for developing IFI. Survival was lower in patients with an invasive fungal disease, vascular complications, and renal dysfunction within the first 100-days post-transplant.

**Conclusions:**

The use of antifungal prophylaxis and transplantation with a deceased donor were protective factors for IFI, whereas reoperation and renal replacement therapy were risk factors, and the occurrence of and IFI diminishes post-transplant survival.

## Introduction

Solid Organ Transplants (SOT) provide increased life expectancy and quality of life for various comorbidities of organ failure, with a one-year survival rate of more than 80%^1^ More than half of Liver Transplant (LT) recipients develop some type of infection post-transplant,^2^ mostly of bacterial etiology. However, fungal infections are an important cause of morbidity and mortality after SOT. The highest incidence of Invasive Fungal Infections (IFI) among SOT occurred in liver recipients in a TRANSNET cohort, with 339 cases (31.8%). The emergence of new pathogens with different susceptibility profiles renders it essential to review antifungal prophylaxis protocols in transplant services. In the 1990s, the incidence of IFI varied from 6% to 47% in LT recipients, with high mortality rates^3^^,^^4^ With the advent of better surgical techniques, adjustments in immunosuppression, and preventive strategies such as antifungal prophylaxis, these rates have been reduced, reaching incidence rates as low as 4% in a multicenter American study^1^^,^^5^^,^^6^ The mortality rate from invasive candidiasis in LT is 30.9% according to TRANSNET data^1^

In studies of risk factors for IFI post-LT, several factors were found, with results sometimes differing among studies. The most commonly cited risk factors are prolonged surgical time, reoperation, retransplantation, need for renal replacement therapy, fulminant hepatitis, choledochojejunostomy, *Candida spp.* colonization, extensive transfusions of blood components, and higher MELD score^3^^,^^4^^,^^6^, ^7^, ^8^, ^9^, ^10^, ^11^, ^12^ Studies in different centers identify different risk factors, with no consensus in the literature. Currently, guided antifungal prophylaxis is recommended only for patients considered at high risk for IFI. Some studies have already shown the safety of guided prophylaxis compared to universal prophylaxis^3^^,^^13^^,^^14^

It is important to define which patients will benefit from prophylaxis, determining poorly established risk factors for IFI in the post-LT period to facilitate proper prophylaxis, promoting greater graft and patient survival.

The main objective was to identify the risk factors associated with IFI in the first 100-days after LT, as well as to identify the risk factors associated with death in the studied cohort in the first 100-days after LT.

## Methods

This is a retrospective multicenter case-control study conducted in two liver transplant reference hospitals in the city of São Paulo: Hospital das Clínicas da FMUSP (HCFMUSP) and Hospital Israelita Albert Einstein ‒ Unidade Vila Santa Catarina (HIAE-VSC). We included patients undergoing LT from January 2002 to December 2017 presenting with an IFI diagnosed within the first 100-days after transplantation, according to the definitions published by the EORTC in 2020^15^ Patients who died within the first 48-hours after liver transplantation and IFI with positive cultures collected intraoperatively or within the first 24-hours after transplantation were excluded.

### Control group

For each case included in the study, three controls were randomly selected from the cohort of patients who underwent LT at the corresponding hospital in the same period of time, without matching criteria.

### Protocols

At HCFMUSP, the immunosuppression protocol consists of methylprednisolone or basiliximab at anesthetic induction, with subsequent progressive dose reduction, followed by prednisone for 3‒6 months post-transplant. Tacrolimus is initiated on the second day after transplantation, with target serum levels maintained around 8 to 10 mg/dL. Mycophenolate mofetil or sodium is introduced if there is a risk of renal dysfunction or a higher risk of chronic kidney disease (advanced age and/or diabetes). Antibiotic prophylaxis includes ampicillin and cefotaxime or amikacin for 48-hours, with adjustments according to the recipient's previous or donor's current infections. Prophylaxis for strongyloidiasis consists of ivermectin 6 mcg/kg/day for 2-days post-transplant. Patients at high risk for Cytomegalovirus (CMV) receive prophylaxis with ganciclovir 5mg/day or valganciclovir 900mg/day for 3-months, in patients with normal renal function. Others undergo surveillance with quantitative PCR, with preemptive treatment as necessary. The antifungal prophylaxis protocol changed over time. There were 3-periods with different prophylaxis regimens: conventional amphotericin B for high-risk patients (2002 to 2006); conventional amphotericin B for high risk and fluconazole for intermediate-risk patients (2007 to 2009); fluconazole for intermediate to high-risk patients (2010 to 2017). Fluconazole is administered at a daily dose of 400 mg, and amphotericin B at a dosage of 0.7 mg/kg for 21-days for patients with normal renal function. Patients at high risk include those with fulminant hepatitis, undergoing retransplantation, or requiring Renal Replacement Therapy (RRT) during transplantation or in the subsequent 7-days. Patients with two or more of the following factors are considered intermediate risk: reoperation, antibiotics in the 30-days pre-transplant, ICU admission in the 30-days pre-transplant, antibiotic prophylaxis for spontaneous bacterial peritonitis, choledochojejunostomy, or *Candida spp.* colonization in two or more sites.

At HIAE-VSC, the immunosuppression protocol includes hydrocortisone at anesthetic induction, followed by hydrocortisone or intravenous mycophenolate sodium, transitioning to prednisone with progressive dose reduction for 3‒6 months post-transplant. Tacrolimus is initiated on the second day post-transplantation. Mycophenolate mofetil or sodium is initiated if there is a risk of renal dysfunction or higher risk of chronic kidney disease (such as advanced age and/or diabetes). Antibiotic prophylaxis consists of amoxicillin with clavulanate and cefotaxime for 48-hours, adjusted according to the recipient's previous or donor's current infections. Prophylaxis for strongyloidiasis and CMV are the same as at HCFMUSP. The antifungal prophylaxis protocol is fluconazole for 21-days for patients with risk factors such as fulminant hepatitis, retransplantation and renal replacement therapy.

### Data collection

Data collection at HCFMUSP was carried out by consulting the prospectively maintained database of the LT service at HCFMUSP and through the review of institution electronic records. Data collection at the Hospital Israelita Albert Einstein (Vila Santa Catarina Unit) was done through the review of prospectively maintained data records by the LT service at HIAE-VSC, as well as institution physical and electronic records. All data was stored in a database on REDCap®.

The collected dichotomic variables were: gender, underlying liver disease, IFI agent and site of infection, retransplantation, prophylaxis for spontaneous bacterial peritonitis, post-transplant renal dysfunction, post-transplant renal replacement therapy, use of antifungal prophylaxis, antifungal prophylaxis regimen used, broad-spectrum antibiotic use in the 30-days prior to transplantation, *Candida spp.* colonization in the 90-days prior to transplantation, biliary anastomosis, reoperation, acute rejection episode, graft vascular complications, primary graft dysfunction (defined by Olthoff Criteria),^16^ CMV infection (defined by serum PCR > 1000 IU/mL or progressive elevation of serum PCR value or biopsy evidence of target organ injury or physician-initiated therapy), and death. The continuous variables analyzed included age, surgery duration, cold ischemia time, functional MELD, pre-transplant hospital stay duration, post-transplant ICU stay duration, number of blood concentrates during transplant. Variables related to exposure were collected for cases until the IFI event and for controls until the end of follow up (100-days after transplantation).

### Definitions

Invasive fungal disease was defined as a positive culture in sterile material, associated with criteria previously published by EORTC/MSG in 2020 for proven or probable aspergillosis^15^ The project was developed and initiated before the publication of this new criteria for IFI by EORTC/MSGERC but the cases remained within the criteria. Early graft dysfunction was defined according to Olthoff Criteria (presence of one or more of the following: total bilirubin ≥ 10mg/dL on the seventh postoperative day; INR ≥ 1.6 on the seventh postoperative day; ALT or AST > 2,000 IU/L in the first 7-days post-transplant)^16^ Renal dysfunction was defined according to KDIGO criteria^17^

### Statistical analysis

A comparison of clinical and demographic characteristics between cases and controls was conducted. Chi-Square test or Fisher test was used for categorical variables, and Student's *t*-test or Mann-Whitney-Wilcoxon test for continuous variables. A 95% Confidence Interval was considered. The primary outcome was the occurrence of IFI within 100-days post-transplant. Secondary outcome was mortality within the first 100-days post-transplant. For the bivariate analysis of risk factors for the development of IFI, the chi-square test or Fisher's exact test was conducted for dichotomic variables, and the Mann-Whitney test for continuous variables. Multivariate analysis was carried out through stepwise logistic regression. For the outcome of death within the first 100-days post-transplant, bivariate and multivariate analyses were performed using Cox regression analysis. The proportional hazard assumption was tested for the variables included in the analysis using Schoenfeld residual plots. The inclusion criterion in the multivariate analysis for both outcomes was p < 0.1 in the bivariate analysis. Variables that decreased the logarithmic probability of −2 or had p < < 0.05 were retained in the model. The following independent variables were considered time-depend on survival analysis: reoperation, acute rejection episode, graft vascular complications, IFI and CMV infection. The data were processed in Stata 17.

### Ethical considerations

The project was approved by the Medical Ethics Committee of both centers (HCFMUSP, CAAE: 25932319.1.1001.0068 and HIAE-VSC, CAAE: 25932319.1.2003.0071). As it is a retrospective study of medical record review, without interventions, the requirement for informed consent was waived. The entire process was carried out confidentially, ensuring the subjects' data security.

## Results

**Fig. 1:**
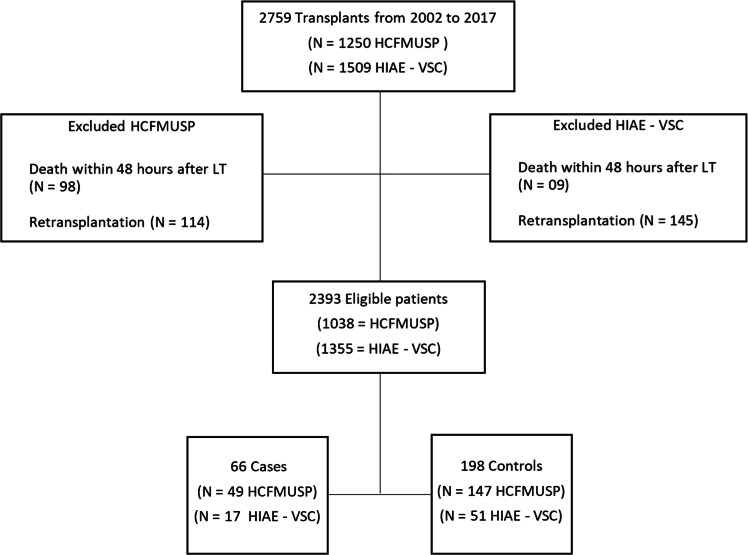
Flowchart for identifying cases and controls. HCFMUSP, Hospital das Clínicas da Faculdade de Medicina da Universidade de São Paulo; HIAE-VSC, Hospital Israelita Albert Einstein Vila Santa Catarina.

From 2002 to 2017, a total of 2759 liver transplants were performed in both centers. We excluded 107 patients who died within 48-hours post-transplant and 259 retransplant records, resulting in 2393 eligible subjects for the study. Cultures identified 66 cases and 198 controls across both centers, as depicted in Fig. 1 outlining patient inclusion and exclusion criteria.

Clinical and demographic data of study cases and controls are presented in Table 1. Most patients were male in both groups. Median age was 51-years for cases and 54-years for controls. Median MELD scores were 21 for cases and 20 for controls. The most common causes of cirrhosis were chronic hepatitis C (34.9% of IFI cases) and alcoholic cirrhosis (19.7%). Hepatocellular carcinoma was present in 33% of IFI cases, with 4.5% of transplants were due to fulminant hepatitis. Three percent involved simultaneous liver-kidney transplants. Significant differences between groups were noted in median length of hospital stay and ICU admission: 42 and 12.5-days for cases, compared to 18.5 and 5-days for controls, respectively.

**Table 1:** Clinical and demographic characteristics of individuals with invasive fungal disease and controls in the two centers (HCFMUSP e HIAE-VSC), 2002‒2017.

	IFI (n = 66)	Controls (n = 198)	p-value
**Age, median (IQR)**	51 (20 – 72)	54 (15 – 73)	0.835
**Male gender**	47 (71.2)	121 (61.1)	0.140
**Center**			1.000
HCFMUSP	49 (74.2)	147 (74.2)	
HIAE-VSC	17 (25.8)	51 (25.8)	
**Functional MELD, median (IQR)**	21 (7 – 40)	20 (5 – 40)	0.190
**Median (IQR) length of stay (days)**	42 (4 – 149)	18.5 (3 – 256)	0.000
**Median (IQR) ICU length stay (days)**	12.5 (2 – 100)	5 (0 – 100)	0.000
**Disease**			
HCV	23 (34.9)	64 (32.3)	0.705
HBV	07 (10.6)	17 (8.6)	0.621
Alcoholic hepatites	13 (19.7)	39 (19.7)	1.000
Cryptogenic	10 (15.2)	24 (12.1)	0.524
Autoimmune	2 (3)	10 (5)	0.495
Others	25 (37.9)	80 (40.4)	0.635
Retransplantation	08 (12.1)	11 (5.6)	0.074
Fulminant	3 (4.6)	13 (6.6)	0.551
Hepatocellular carcinoma	22 (34.4)	62 (31.3)	0.648
Liver kidney transplant	2 (4.1)	06 (4.1)	0.993
Deceased donor	58 (87.9)	187 (94.4)	0.074
Pre transplant SBP profhylaxis	12 (20.3)	29 (15.1)	0.341
Pre transplant antimicrobial use	16 (28.1)	42 (22)	0.341
Surgical duration (hours) median (IQR)	7 (2 – 15)	7 (3 – 36)	0.274
Cold isquemia duration (hours) median (IQR)	7 (1 – 15)	7 (1 – 16)	0.611
Biliodigestive diversion anastomosis	5 (7.9)	22 (11.8)	0.397
Red blood cells (median) (IQR)	2 (0 – 15)	1 (0 – 12)	0.109
Vascular complications	13 (20.6)	21 (10.8)	0.046
Kidney dysfunction	54 (84.4)	111 (56.6)	0.000
Hemodialysis	39	62	0.000
Acute rejection	15 (23.4)	43 (22.6)	0.894
Reoperation	38 (59.4)	37 (19)	0.000
Colonization by *Candida sp*.	4 (6.15)	11 (5.6)	0.864
Antifungal prophylaxis	39 (59.1)	107 (54.6)	0.524
Cytomegalovirus infection	9 (13.9)	27 (14.8)	0.989
Primary non function	13 (20.6)	21 (11.2)	0.057
Early graft dysfunction (Olthoff)	35 (53.9)	87 (44.6)	0.197

IQR, Interquartil Interval; HCFMUSP, Hospital das Clinicas da Faculdade de Medicina da Universidade de São Paulo; HIAE-VSC, Hospital Israelite Albert Einstein – Unidade Vila Santa Catarina; ICU, Intensive Care Unit; HCV, Viral Hepatitis C; HBV, Viral Hepatitis B; SBP, Spontaneous Bacterial Peritonitis; DFI, Invasive Fungal Disease; MELD, Model for End-stage Liver Disease.

Among the 66 IFI cases, 21 (31.8%) resulted in death due to IFI, according to medical record data. Of 264 evaluated patients, 146 received antifungal prophylaxis, predominantly fluconazole (67.8% of cases). No difference was observed between fluconazole and amphotericin B prophylaxis. Among 101 patients undergoing RRT, 7/101 (6.9%) developed IFI without antifungal prophylaxis; 25/101 (24.8%) patients developed IFI despite prophylaxis, with no difference in IFI rate noted among different antifungal agents.

Table 2 details fungal agents and infection sites. *Candida spp.* predominated (58-cases), mostly non-albicans (57.6%), followed by *Candida albicans* (30.3%). Filamentous fungi included *Aspergillus spp.* (10.6%) and one *Mucor spp*. infection. Intrabdominal was the primary infection site (54%), followed by bloodstream infections (27%).

**Table 2:** Agents and sites of the first episode of early invasive fungal disease in both centers (HCFMUSP e HIAE-VSC), 2002‒2017.

Agents	n (%)
***Candida sp****.*	58 (87.9)
*C. non albicans*	38 (57.6)
*C. glabrata*	14
*C. parapsilosis*	7
*C. tropicalis*	6
*C. krusei*	5
*C. peliculosa*	2
*C. dublienensis*	2
*C. guilliermondii*	1
*C. pseudotropicalis*	1
*C. albicans*	20 (30.3)
***Aspergillus sp.***	7 (10.6)
***Mucor sp.***	1 (1.5)
	**66 (100)**
**Sites**	**n (%)**
Intrabdominal	40 (54)
Bloodstream	20 (27.0)
Pulmonary	10 (13.5)
Soft parts	04 (5.4)
	**74 (100)**

**Fig. 2:**
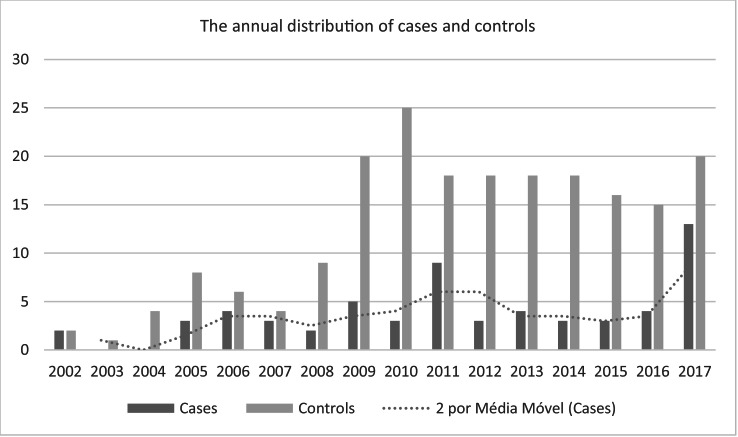
The annual distribution of cases and controls.

The distribution of IFI cases from 2002 to 2017 showed no statistical difference across years (Fig. 2, p p = 0.346).

Bivariate analysis identified several risk factors for IFI occurrence (Table 3), including antifungal prophylaxis, deceased donor, reoperation, renal replacement therapy, MELD > 30, antibiotic use, retransplantation, hospital stay > 30-days, graft vascular complications, and primary graft non-function. Dialytic renal dysfunction and early reoperation within 100-days were independent risk factors for IFI occurrence. Antifungal prophylaxis and deceased donor status were protective against IFI.

**Table 3:** Univariate and multivariate analysis of risk factors for invasive fungal disease in the two centers (HCFMUSP and HIAE-VSC), 2002‒2017.

	OR	p-value	Confidence Interval	OR	p-value	Confidence Interval
Gender	1.57	0.14	0.86 – 2.88	0.549	0.094	0.272 – 1.107
Center	1.00	>0.99	0.53 – 1.89			
Pre transplant SBP prophylaxis	1.44	0.34	0.68 – 3.03			
Pre transplant antimicrobial use (30-days)	6.30	<0.001	2.60 – 15.29			
MELD (> 30)	2.10	0.01	1.16 – 3.79			
Fulminant hepatites	0.68	0.77	0.19 – 49.15			
Retransplantation	2.35	0.07	0.90 – 6.11			
Hepatocelliular carcinoma	1.15	0.21	0.63 – 209			
Length of stay (> 30-days)	0.36	0.01	0.16 – 0.81			
UCI Length of stay (> 15-days)	1.15	0.74	0.51 – 2.62			
Deceased donor	0.43	0.07	0.16 – 1.11	0.312	0.030	0.109 – 0.893
Surgical duration (> 6-hours)	1.17	0.58	0.68 – 2.02			
Cold isquemia duration (> 8-hours)	1.04	0.92	0.54 – 1.98			
Biliodigestive diversion anastomosis	0.65	0.49	0.23 - 1.79			
Kidney dysfunction pos LT	4.14	<0.001	1.99 – 8.59			
Hemodialysis pos LT	3.37	<0.001	1.88 – 6.06	4.104	0.001	1.744 – 9.656
Acute rejection	1.05	0.89	0.54 – 2.05			
Reoperation	6.24	<0.001	3.38 – 11.53	5.368	0.000	2.739 – 10.521
Vascular disorder	2.14	0.05	1.00 – 4.58			
Primary non function	2.07	0.06	0.97 – 4.42			
Early graft dysfunction (Olthoff)	1.61	0.10	0.90 – 2.86			
Colonization by *Candida sp*. 90-days pre transplant	1.13	0.77	0.35 – 3.66			
Antifungal prophylaxis	2.74	<0.001	1.58 – 4.77	0.397	0.036	0.167 – 0.941
Antifungal prophylaxis drugs						
Amphotericin B	0.32	0.46	0.04 – 2.60			
Fluconazole	1.66	0.08	0.94 – 2.91			
Cytomegalovirus infection	1.00	0.99	0.45 – 2.27			

IQR, Interquartile Range; HCFMUSP, Hospital das Clínicas of the Faculty of Medicine of the University of São Paulo; OR, Odds Ratio; HIAE-VSC, Hospital Israelite Albert Einstein – Vila Santa Catarina Unit; ICU, Intensive Care Unit; HCV, Viral Hepatitis C; HBV, Viral Hepatitis B; SBP, Spontaneous Bacterial Peritonitis; DFI, Invasive fungal Disease; MELD, Model for End-stage Liver Disease; LT, Liver Transplant.

Bivariate analysis for death within 100-days post-transplant identified significant risk factors: IFI development, MELD > 30, ≥ 5 red blood cell transfusions, renal dysfunction with or without dialysis, vascular complications, primary graft non-function, and early graft dysfunction. Multivariate analysis identified IFI, vascular complications, and renal dysfunction within the first 100-days post-transplant as independent risk factors for mortality (Table 4).

**Table 4:** Univariate and multivariate analysis of risk factors for death in the two centers (HCFMUSP and HIAE-VSC), 2002‒2017.

	HR	p-value	Confidence Interval	HR	p-value	Confidence Interval
Age	1.013	0.197	0.993 – 1.034			
Gender	0.783	0.404	0.441 – 1391			
IFI development	10.484	0.000	5.865 – 18.743	8.282	0.000	4.548 – 15.084
Pre transplant SBP prophylaxis	1.666	0.122	0.872 – 3.183			
Funcional MELD (> 30)	2.318	0.002	1.351 – 3.977	1.020	0.137	0.994 – 1.047
Hepatocellular carcinoma	1.125	0.685	0.636 – 1.993			
Fulminant hepatitis	0.606	0.487	0.148 – 2.487			
Liver-kidney transplant	1.051	0.945	0.255 – 4.338			
Retransplantation	2.722	0.886	0.426 – 2.685			
Length of stay (> 30-days)	3.797	<0.001	2.210 – 6.522			
UCI Length of stay (> 15-days)	1.966	0.01	1.149 – 3.364			
Deceased donor	0.592	0.226	0.253 – 1.383			
Surgical duration (> 6-hours)	0.962	0.90	0.523 – 1.768			
Cold isquemia duration (> 8 h)	1.152	0.610	0.667 – 1.992			
Biliodigestive diversion anastomosis	0.899	0.821	0.357 – 2.264			
Red blood cells (> 5)	1.122	0.007	1.033 – 1.220			
Kidney dysfunction 100-days after transplant	8.414	0.000	3.038 – 23.303	3.979	0.011	1.372 – 11.542
Acute rejection	0.584	1.162	0.275 – 1.241			
Reoperation	3.448	0.000	2.014 – 5.903			
Vascular disorder 100 days after transplant	3.531	0.000	1.957 – 6.370	3.858	0.000	2.099 – 7.093
Primary non function	2.549	0.003	1.360 – 4.777	1.725	0.094	0.911–3.269
Early graft dysfunction (Olthoff)	2.167	0.006	1.247 – 3.766			
Cytomegalovirus infection	0.88	0.75	0.40 – 1.94			

HR, Hazard Ratio; ICU, Intensive Care Unit; SBP, Spontaneous Bacterial Peritonitis; IFI, Invasive Fungal Infection; CH, Red blood cell concentrate; MELD, Model for End-stage Liver Disease.

## Discussion

Identifying risk factors associated with the development of IFI is crucial for implementing appropriate screening and tailored use of antifungal prophylaxis, thereby reducing morbidity and mortality following LT. In our study across two major LT centers in São Paulo, risk factors for IFI occurrence within 100-days post-transplant were identified as renal replacement therapy and reoperation. Protective factors included the use of antifungal prophylaxis and deceased donor transplantation. Regarding mortality within the same period, IFI occurrence, graft vascular disorders, and renal dysfunction were identified as worse prognostic factors.

Renal replacement therapy as a risk fator for IFI occurrence is accordance to previously studies (colocar as referencias Gianella, Melenotte e Fortun). In our cohort, 101 subjects underwent renal replacement therapy, and 32 IFI cases were identified in this population, with only 7 not receiving prophylaxis. Despite prophylactic antifungal use, breakthrough infections occurred in 25-cases. Cases of IFI occurring despite fluconazole prophylaxis may have occurred by potential variability in serum levels during renal replacement therapy, affecting drug pharmacokinetics and pharmacodynamics^18^

Reoperation was also identified as an independent risk factor for IFI. In a literature review, reoperation and laparotomy are associated with up to a 10-fold increase in IFI risk^19^ In an Australian case-control study investigating early IFI risk factors, reoperation was also independently associated in multivariate analysis, along with biliary-enteric anastomosis, and intraoperative transfusion of ≥40-units. Intestinal manipulation during reoperations or retransplantation predisposes to translocation of *Candida* species into the peritoneal cavity or bloodstream^13^ In our center, reoperation is considered a minor criterion for initiating antifungal prophylaxis; however, this study underscores its importance as a significant risk factor, and resulted in a protocol modification, and we have included reoperation as a major criterion for antifungal prophylaxis.

Contrary to the literature, our study did not identify fulminant hepatitis and retransplantation as independent risk factors for IFI, despite these being classically associated factors^4^^,^^6^^,^^14^^,^^20^ Retransplantation can increase the risk of invasive aspergillosis up to 29-fold^19^ Both participating centers consider these indications for prophylaxis. Therefore, the use of prophylaxis in our patients diagnosed with fulminant hepatitis and retransplantation may have proven effective in preventing IFI development in our cohort.

Regarding antifungal prophylaxis, Neyra et al.'s cohort did not find a difference in IFI incidence between high-risk patients receiving or not receiving prophylaxis (fluconazole or micafungin). The number needed to treat to prevent one IFI case was 105, however, IFI cases in this sample were small^21^ Winston et al. conducted a randomized clinical trial comparing anidulafungin and fluconazole in high-risk IFI patients, finding no significant difference, though no cases of aspergillosis occurred in the anidulafungin group. “Breakthrough” cases in the fluconazole group included 5 out of 8 *Candida spp*. isolates resistant to the drug^9^ Kang et al., in a study across 5 centers in South Korea, found no inferiority in micafungin prophylaxis compared to fluconazole universal prophylaxis in LDLT^22^ However, Fortun et al., in a multicenter study, compared caspofungin and fluconazole, identifying superiority in preventing breakthroughs in the caspofungin group, with no difference in mortality^10^ These findings are reinforced by a meta-analysis by Gatti et al., demonstrating non-inferiority of echinocandins, but a higher tendency toward breakthroughs in echinocandin groups in renal replacement therapy patients when excluding the previously mentioned Fortun et al. study^23^

The importance of studies comparing echinocandins with fluconazole use arises in the context of emerging *Candida spp*. strains resistant to fluconazole, as well as their lack of interaction with calcineurin inhibitors and potential activity against aspergillosis. The exclusively intravenous presentation and high cost are limitations for routine echinocandin use in IFI prevention. The incidence of *Aspergillus spp.* and other filamentous fungi infections is relatively low in these studies^24^^,^^25^ Literature consensus supports *Candida spp*. prophylaxis in high-risk patients, though drug, dosage, and duration are debated. For instance, fluconazole use varies from 21 to 60-days, at doses ranging from 100 to 400 mg^20^ In this cohort, *Candida spp*. were the main identified agents, primarily affecting the intra-abdominal site, consistent with other literature series^3^ The incidence of *Aspergillus spp.* infection in our two evaluated centers was low, with most occurring during a period of construction work in the hospital. Based on our study results, maintaining the current service antifungal prophylaxis protocol appears safe.

In our study, deceased donor transplantation was also identified as a protective factor against IFI, suggesting that Living Donor Transplantation (LDLT) may be associated with increased fungal infection risk. In our cohort, 19 LDLT cases were performed, with 42.1% developing IFI. The increased risk of IFI in LDLT may be associated with surgical complexity. In this technique, the graft is smaller with narrower blood vessel and bile duct diameters, potentially increasing the risk of biliary complications such as fistulas and strictures^3^^,^^22^^,^^26^ Due to the small number of living-donor recipients in this sample, the analysis of this finding may be overestimated. Therefore, studies including a larger population of LDLT are needed to better evaluate this finding. These are under-recognized risk factors in the literature and warrant further study and consideration for prophylactic measures in this setting.

In this cohort, controls weren’t selected through matched randomization. Although no statistically significant differences were observed between the groups, this should be considered a limitation of the study. Additionally, the small number of living-donor transplants in this cohort may have influenced the observed findings.

The main limitations of this study include the small number of fungal infections; a larger cohort might have identified different risk factors. Fungal infections identified exclusively by anatomopathological examination were also not included. The method used to identify IFI cases relied on positive cultures meeting the 2020 EORTC criteria, this choice excluded other IFI cases diagnosed with indirect evidence that could meet probable or possible criteria, such as invasive aspergillosis diagnosed by galactomannan or cryptococcosis diagnosed by latex agglutination. Infections like pneumocystosis, not commonly identified via cultures, were also excluded.

Another limitation is its retrospective nature, retrieving data from 2002 onwards, a time when routine antifungal sensitivity testing was not performed, precluding assessment of whether fungal disease cases occurring in patients on prophylaxis were associated with resistance patterns.

## Conclusions

We identified that the use of antifungal prophylaxis and deceased donor were protective factors against IFI development within the first 100-days post-transplant. No superiority was observed between the regimens used. The lack of superiority among prophylactic regimens is consistent with the predominance of *Candida spp*. as the main causative agents, rather than filamentous fungi. Breakthrough infections occurred mainly in patients under renal replacement therapy, possibly due to underdosing of antifungals.

We observed that *Candida spp.* were the primary agents of IFI. When aggregating all non-albicans *Candida* species, they were the most frequently identified; however, upon separate species evaluation, *C. albicans* was the most isolated species. The main site of invasive fungal disease was the intra-abdominal site. The data from our cohort are consistent with series found in the literature.

The renal replacement therapy and reoperation were considered risk factors. Regarding mortality, IFI was a risk factor within the first 100-days post-liver transplant, as well as vascular graft complications and renal dysfunction.

## Abbreviatures

ALT, Alanine Aminotransferase; AST, Aspartate Aminotransferase; CMV, Cytomegalovirus; EORTC/MSGERC, European Organization for Research and Treatment of Cancer and the Mycoses Study Group Education and Researh Consortium; FMUSP, *Faculdade de Medicina da Universidade de São Paulo*; HBV, Viral Hepatitis B; HCFMUSP, *Hospital das Clínicas da Faculdade de Medicina da Universidade de São Paulo*; HCV, Viral Hepatitis C; HIAE-VSC, Hospital Israelita Albert Einstein ‒ Unidade Vila Santa Catarina; HR, Hazard Ratio; IC, Confidence Interval; ICU, Intensive Care Unit; IFI, Invasive Fungal Infections; IQR, Interquartile Interval; LT, Liver Transplant; MELD, Model for End-Stage Liver Disease; OR Odds Ratio; PCR, Polymerase Chain Reaction; RR, RRT, Relative Risk Renal Replacement Therapy; LDLT, Living Donor Transplantation; SBP, Spontaneous Bacterial Peritonitis; SOT Solid Organ Transplants; UI, Units.

## Data availability

Data must be requested from the corresponding author.

## Authors’ contributions

All authors contributed to the study conception and design. Material preparation, data collection, and analysis were performed by Larissa N de A Gouveia, Bruno G P Romeo and Julia Rezende. Analysis was performed by Maristela P. Freire, Alice T W Song and Larissa N de A Gouveia. The first draft of the manuscript was written by Larissa N de A Gouveia, and all authors commented on previous versions of the manuscript. All authors read and approved the final manuscript.

## Funding

There was no funding for this work.

## Conflicts of interest

The authors declare no conflicts of interest.
